# Semiconductor Multimaterial Optical Fibers for Biomedical Applications

**DOI:** 10.3390/bios12100882

**Published:** 2022-10-17

**Authors:** Lingyu Shen, Chuanxin Teng, Zhuo Wang, Hongyi Bai, Santosh Kumar, Rui Min

**Affiliations:** 1 Center for Cognition and Neuroergonomics, State Key Laboratory of Cognitive Neuroscience and Learning, Beijing Normal University, Zhuhai 519087, China; 2 Guangxi Key Laboratory of Optoelectronic Information Processing, School of Optoelectronic Engineering, Guilin University of Electronic Technology, Guilin 541004, China; 3 College of Electronics and Engineering, Heilongjiang University, Harbin 150080, China; 4 Shandong Key Laboratory of Optical Communication Science and Technology, School of Physics Science and Information Technology, Liaocheng University, Liaocheng 252059, China

**Keywords:** multimaterial optical fibers, semiconductor optical fibers, biomedical applications, optical fiber fabrication

## Abstract

Integrated sensors and transmitters of a wide variety of human physiological indicators have recently emerged in the form of multimaterial optical fibers. The methods utilized in the manufacture of optical fibers facilitate the use of a wide range of functional elements in microscale optical fibers with an extensive variety of structures. This article presents an overview and review of semiconductor multimaterial optical fibers, their fabrication and postprocessing techniques, different geometries, and integration in devices that can be further utilized in biomedical applications. Semiconductor optical fiber sensors and fiber lasers for body temperature regulation, in vivo detection, volatile organic compound detection, and medical surgery will be discussed.

## 1. Introduction

Respiration rate, heart rate, foot pressure, and joint mobility are crucial in diagnosing health issues and abnormal occurrences such as tachypnea, hypoxemia, tachycardia, and bradycardia [1,2]. Different sensors have been utilized to gather physiological indications and body movement signals, especially electronic device-based sensors, which are critical for measuring parameters such as pulse beat [3], heart rate [4], and finger bending [5]. Electricity sensing has apparent limitations, such as a small linear response interval, electrical safety difficulties, and electromagnetic interference (EMI) [6]. These limitations drive researchers to explore new functionalities of silica glass fibers. Silica glass fibers, which include the coprocessing of several families of materials into a single fiber [7,8], have been utilized by the telecommunications industry [9,10,11,12]. The search for novel materials [13] for this platform is being driven by the need to extend the functionality of fibers to achieve wider transmission windows and to improve their nonlinear performance [14,15,16,17,18]. A broad variety of materials, including polymers [19,20], gases [21], liquid crystals [22], and even soft glasses [23], have been infiltrated into microstructured optical fiber (MOF) templates in the form of fluids. However, due to the growing interest in semiconductor photonics in recent years, several more complex methods have been created to combine more complicated crystalline and amorphous semiconductor materials into fibers [24,25,26,27,28]. Semiconductor optical fibers, despite being susceptible to electromagnetic interference and sacrificing some outstanding properties of silica optical fibers, open doors for the next generation of semiconductor devices [29].

Lab-on-fibers [30,31], which are revolutionary miniaturized sensors extensively employed in biomedicine [32], point-of-care diagnostics [33], and photonic integrated devices [34], are the result of the most recent advancements in fabrication processes for integrating these materials into fiber devices. By enhancing conventional nanolithography, modern methods enable the exact production of optical fiber-tips to precisely control parameters [30]. Various optical nanostructures, such as photonic crystals [35] and plasmonic platforms [30], have been merged with optical fibers using these techniques. The need for creating miniaturized optical biosensors drove the first proof of concept demonstrations [36,37], uncovering massive possibilities for biomedical compounds recognition [31], such as prostate-specific antigen for prostate cancer recognition [38] and thyroglobulin for thyroid cancer recognition [36].

The selection of the coating’s composition materials can generate intriguing phenomena, which may lead to the development of a wide variety of sensors. Lossy mode resonance (LMR) results from the interaction of light propagating through a fiber covered with a coating that possesses the necessary optical characteristics [39]. This effect generates an absorption maximum at the optimal wavelength. LMR can be supported by fabricating films from several materials [40], including indium tin oxide (ITO) [41], zinc oxide [42], polymers [43], silicon nitride [44], etc. Sensors with different functionalities can be fabricated. For instance, hydrogen gas sensors can be fabricated using ITO nanoparticle coating [41], sulfide gas sensors can be fabricated using zinc oxide nanorods coating [45], and PH [46] and relative humidity [43] sensors can be fabricated utilizing polymeric coatings of polyallylamine hydrochloride (PAH) and poly-acrylic acid (PAA). The prevalence and usability of basic fibers stimulate the development of techniques and technologies for integrating photonic devices directly onto them [47].

The pioneering work performed in the 1950s to examine the optical characteristics of a wide variety of materials is considered the beginning of the field of semiconductor photonics [29]. Even though the majority of these early efforts were concentrated on group IV materials, the variety of electrical and optical characteristics that are provided by the broader range of semiconducting materials presents an enormous space to construct devices [48,49]. Silicon continues to be the material that is most often utilized in integrated photonic circuits due to its high transparency level, significant nonlinear coefficient [50], and high threshold for optical damage. Because of these features, silicon core fibers may exhibit outstanding behaviors in the fields of nonlinear optical applications and optoelectronics (OE) [51]. However, germanium is receiving greater attention in the mid-infrared for applications in life science [52]. The most widely employed techniques to produce semiconductors include Czochralski (CZ) growth [53], float zone (FZ) growth [54], laser-heated pedestal growth (LHPG) [55], liquid phase epitaxy (LPE) [56], and traveling solvent FZ [57]. Compound semiconductors, such as II-VIs and III-Vs, are preferred to simple semiconductors for light production and electro-optic modulation. Although work in the subject of semiconductor optical fibers is still in its relative infancy, it already encompasses a variety of unary and compound materials [29].

For semiconductor multimaterial optical fibers, the most common fiber geometries include semiconductor core fibers, metal-semiconductor-insulator fibers, MOFs, and photonic bandgap structures (PBGs). The majority of the fibers are fabricated utilizing the molten core method (MCM) and high-pressure chemical vapor disposition (HPCVD). In MCM, a fluid melt is confined by a glass cladding and pulled to fiber dimensions. Both unary semiconductors and compound semiconductors have been researched, with manufacturing lengths commonly approaching hundreds of meters, restricted by preform size [51]. However, polycrystalline as-drawn fibers are inferior for many uses. Crystalline disorder and impurity segregation at grain boundaries generate optical and electrical flaws, scattering and absorptive losses, mechanical weakness, and short carrier lifetimes. Consequently, postprocessing techniques are required to enhance fiber performance, including thermal annealing [58], rapid photothermal annealing (RPP) [59], the interfacial modifier method [60], and laser treatment [61].

The electrical, OE, thermoelectric (TE), and nonlinear optical capabilities of semiconductors are among the most advanced of all materials [61,62,63]. Expanding the use of optical fibers in biomedical applications is made possible by incorporating semiconductors within the fiber. Glass claddings of semiconductor optical fibers are not, however, appropriate for use inside the human body. Therefore, biocompatible materials [64,65] may play an essential role in the development of biomedical semiconductor devices. It is envisaged that enhanced optical fibers would represent the successors of optical probes as tools for sensing [66] and less invasive surgical instruments [67]. Electronic and optical fibers are also capable of building a range of sensor networks, such as those utilized in interior structures and composites for health monitoring [68]. These sensor networks may be formed using several applications. In addition, these networks have the potential to be the building blocks for the next generation of intelligent textiles, in which additional functionality will be generated from the optical fibers themselves rather than via the implantation of point devices [69].

**Figure 1:**
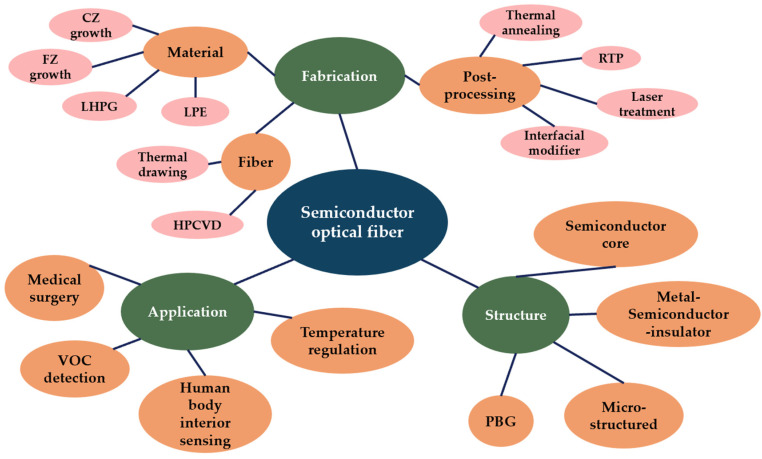
A summary of this review about biomedical semiconductor optical fibers.

In this paper, we review semiconductor multifunctional optical fiber fabrication and biomedical applications. In Section 2, methods for producing semiconducting materials and semiconductor optical fibers and for enhancing fiber performance are introduced. In Section 3, we cover the most common semiconductor optical fibers of different structures, including semiconductor core fibers, metal-semiconductor-insulator fibers, MOFs, and PBGs. In Section 4, biomedical applications of semiconductor multimaterial fibers, including human body temperature regulation, in vivo lesion detection, volatile organic compound (VOC) detection, and neurosurgery lasers, are discussed. Figure 1 shows a summary of this review of biomedical semiconductor optical fibers.

## 2. Fabrication of Semiconductor Optical Fibers

Because of their thermal and mechanical mismatch with typical glass materials, the fabrication of optical fibers from semiconductors is a considerable challenge for the field of materials science [70]. As a result, the platform for optical fibers has been restricted to materials that are consistent with the typical fiber drawing techniques for an extended period. In 2006, a chemical deposition approach was used to create the initial example of a crystalline semiconductor optical fiber [25]. As a result of this endeavor, different methods have been utilized in the fabrication of semiconductor optical fibers. Each of these methods has its own set of benefits and drawbacks. However, since there have been no standardized techniques for fabricating these fibers, their production is relatively costly. This section provides a review of these various methods of fabrication and evaluates the relative benefits of each approach.

### 2.1. Material Fabrication

The production of fabrication begins with material fabrication. Commonly used methods for fabricating semiconductors, such as silicon, germanium, and selenium, include CZ growth, FZ growth, LHPG, and LPE.

**Figure 2:**
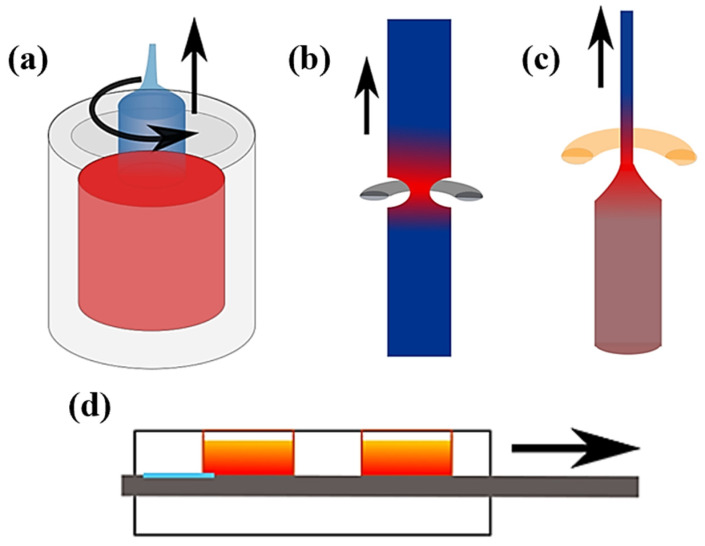
Material fabrication techniques. (Reprinted with permission from ref. [51]. Copyright 2021 Ursula J. Gibson et al.): (**a**) CZ growth, (**b**) FZ growth, (**c**) LHPG, and (**d**) LPE.

CZ growth is the most popular approach to fabricating single-crystal silicon [71]. The process is depicted in Figure 2a. A single crystalline charge is heated over its melting point to form a melt, then a silicon crystal seed is inserted into the melt, and the crystal is progressively removed from the melt and rotated in the opposite direction to the melting crucible [72]. Key factors to control the fabrication process include the rotation rates of the seed and crucible and the pull rate of the seed [73]. In the early phase of CZ growth, the pull rate is large, and the fabricated crystal is small in diameter (3–4 mm), which is dubbed the “neck” of the crystal. CZ growth was first utilized by Dash for producing dislocation-free crystals, and its use has become prevalent in the industry [73].

FZ growth is an alternate and straightforward way of producing a bulk semiconductor crystal of homogeneous composition. If it is possible to create a feed rod with a uniform composition, then a steady-state condition might be created by selecting the growth parameters in such a way that the composition in the melt spontaneously controls the growth temperature [54]. For FZ growth, an inert gas or vacuum atmosphere is employed, and silicon’s surface tension enables a melt zone to be translated vertically down a high-purity rod [51], as depicted in Figure 2b. While reducing convection, magnetic forces stabilize the melt, and temperature gradients may reach tens of Kcm^−1^ [74,75]. During oxide crystal formation in optical furnaces [76], average bulk growth rates are mm per minute, and the greatest FZ temperature gradients are on the scale of 500–1500 Kcm^−1^ [76]. These characteristics are similar to fiber recrystallization. Carbon and oxygen residuals in FZ silicon boules are very small (on the order of 1016 cm^−3^), but in the case of fibers, the existence of oxide cladding may prevent impurities from being reduced to a small amount.

The production of oxide fibers has shown to be the most successful use of LHPG [55], as illustrated in Figure 2c. Temperature gradients for the LHPG technique can reach 104 Kcm^−1^, which is comparable to the values that have been reported for CO_2_ laser treatment of fibers [59,77,78], even though the unconstrained surface may result in undesirable diameter and surface quality variations. In the production of the quartz suspension fibers used as gravity wave detectors [79,80], a more sophisticated version of this optical technology is utilized. This technique is now commercially accessible for the tapering of optical fibers. Tapering stations have been effectively employed in the recrystallization of silicon core fibers [81,82], which has led to the production of crystalline cores with exceptionally high optical quality.

Since its development by Nelson in 1963, LPE has been used to build thin layers of III-V, II-VI, and IV-V1 alloys for material research and device applications [56], as demonstrated in Figure 2d. LPE structures have been utilized to make several electronics and OE. For example, GaAs/GaA1As double-heterostructure (DH) laser diode room-temperature CW lasing [83,84]. CW operation of DH lasers radiating beyond 1 μm was initially accomplished using LPE material in InP/InGaAsP [85], GaAsSb/GaA1AsSb [86], and GaSb/GaA1AsSb [87]. Studies of LPE literature are crucial in systems where the starting semiconductor charge is disproportioned, e.g., pulling InGaSb core fibers [88]. Sb filaments were detected in the as-drawn fibers, and this excess metal functioned as a recrystallization solvent [89]. Intentionally introducing an excess binary system material may produce a crystallization process similar to LPE in a closed vessel where oxidation issues are decreased. Crystal growth rates are often greater with fibers than with LPE, and few studies of a gradient perpendicular to the growth front for the latter method report maximum values of 25 K cm^−1^ [90,91]. Despite varied circumstances, LPE expertise may be applied to the fiber core and used to explore novel materials and solvent combinations for traveling zone expansion.

### 2.2. Semiconductor Optical Fiber Fabrication Process

#### 2.2.1. Thermal Drawing

Thermal drawing is one of the most commonly employed techniques to fabricate semiconductor optical fibers. The fabrication process should meet some of the following requirements. First, cladding materials should possess high viscosity to resist stretching stress. The viscosities and expansion coefficients of amorphous materials should be comparable to prevent deformation of the interface. Second, the temperature window during the fabrication process needs to be large enough to prevent amorphous materials from crystallizing. Last, chemical reactions during thermal drawing must be utilized to create novel materials during fabrication or to prevent fabrication. For example, the reaction between Al and silica can be utilized to create silicon core fibers [92].

**Figure 3:**
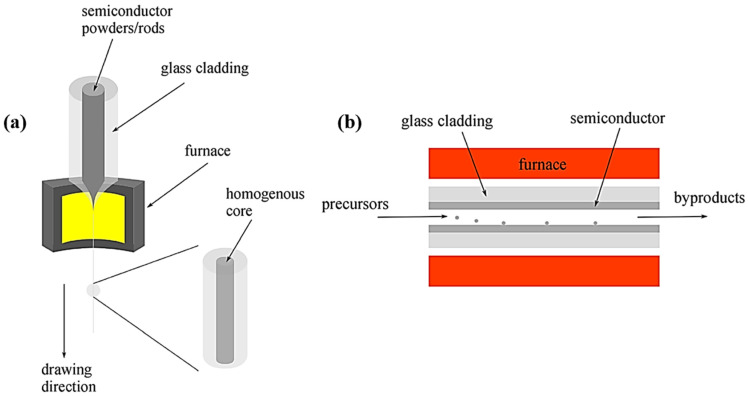
Fiber fabrication techniques. (Reprinted with permission from ref. [93]. Copyright 2021 Hei Chit Leo Tsui et al.): (**a**) thermal drawing and (**b**) HPCVD.

The process is summarized In Figure 3a. A preform is heated using a drawing tower furnace with a temperature that is 50–100 °C higher than the preform cladding glass transition temperature; the preform is drawn by a capstan while the materials within it are softened. Consequently, a fiber with diameter *D_f_* is fabricated. The consequent fiber has the same geometry as the preform. *D_f_* can be adjusted by the preform diameter *D_p_*, preform feeding speed *v_f_*, and drawing speed *v_d_* [14]. This relationship is demonstrated as follows:

(1) $$D_{f}=D_{p}\times \sqrt{\frac{v_{f}}{v_{d}}}$$

A unique technique using drawing towers for producing semiconductor core fibers is referred to as the MCM. The selection criteria for the MCM are similar to the criteria for thermal processing, while semiconductors are confined in cladding material and melt during heating. The MCM can be used to fabricate a variety of unconventional core materials with amorphous or crystalline features. Using the MCM to fabricate semiconductor optical fibers, core materials are placed in a tube that acts as the cladding material; then, through drawing towers, the tube is pulled in a thin robe. After drawing, the core gradually solidifies as the temperature drops; thus, a fiber is fabricated using the MCM. Because of the inconsistent cooling speed, the core might be amorphous or polycrystalline. The light propagation loss in the polycrystalline core optical fiber increases as the scattering loss increases [51]. As a result, postprocessing techniques are needed to enhance the fiber light conducting performance.

#### 2.2.2. High-Pressure Chemical Vapor Deposition

HPCVD is a hybrid method of engineering disciplines to integrate semiconductors and metals into MOFs. HPCVD is currently developed to deposit semiconductor nanowires, coaxial heterojunctions, and other patterned structures inside fibers [66]. High-pressure flow in MPa level can eliminate mass-transport constraints because the fibers’ high tensile strength overcomes the constraints of mass transport [94]. In addition, by considering pores in an MOF template as small chemical reactors, which can be injected into different chemical precursors, it is possible to deposit uniform, thick, and annular deposition on the heated pore walls across lengths ranging from centimeters to meters [95], as shown in Figure 3b.

As the functional materials inside fibers can be chemically deposited in independent stages, enabling fibers to possess design flexibility, fibers fabricated by the HPCVD technique can be further utilized to design fiber-integrated devices, including photoconducting detectors [96], fibers for infrared laser transmission [97,98,99], fibers for thermal sensation [100], radial fiber lasers, and wearable 2D and 3D array light detectors [101]. Although the length of fiber fabricated by HPCVD does not seem to be as scalable as that manufactured by drawing, semiconductor layers of 10 m in length have been accomplished, and larger lengths are conceivable [102]. In addition, HPCVD is extensible for deposition in several fibers using a single high-pressure precursor source, possibly enabling the concurrent fabrication of up to hundreds of fiber devices.

### 2.3. Postprocessing

**Figure 4:**
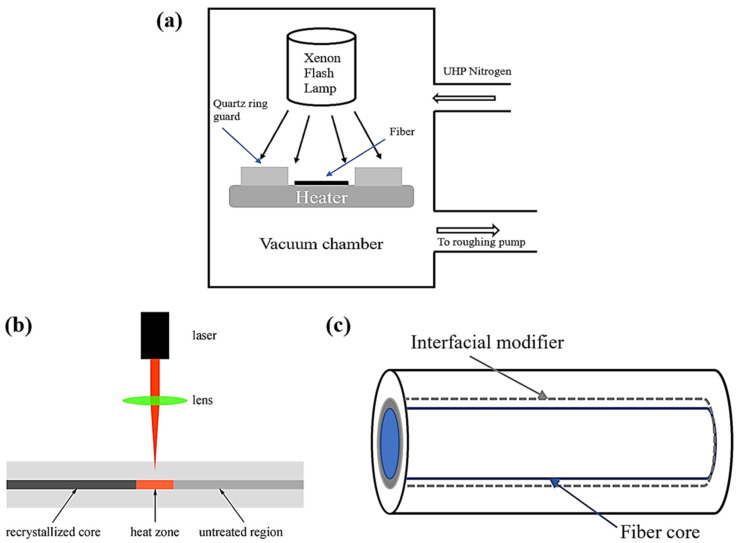
Postprocessing techniques: (**a**) RPP, (**b**) Laser treatment (Reprinted with permission from ref. [93]. Copyright 2021 Hei Chit Leo Tsui et al.), and (**c**) Interfacial modifier.

As the core states are mostly polycrystalline or amorphous in fibers after thermal drawing, postprocessing is frequently necessary to increase the material quality or optimize the fiber shape to fully harness the fibers’ potential. Postprocessing techniques can enhance the performance of semiconductor optical fibers by increasing the crystallinity of semiconductors inside or by reducing element contamination, such as oxide contamination from glass cladding. All postprocessing procedures, with the exception of techniques aimed at reducing element contamination, rely on either thermal annealing or melt-recrystallization to heat a sample [93]. Common techniques used to enhance the multifunctional performance of fibers include thermal annealing [58], RPP [59], the interfacial modifier method [60], and laser treatment [61]. Figure 4 depicts the former postprocessing techniques.

#### 2.3.1. Thermal Annealing

Thermal annealing is a process for controlling the characteristics of materials via crystal formation. During the process, materials do not remelt or recrystallize but rather have controlled nucleation of crystals, during which flaws are removed, and grain sizes are increased [93]. Thermal annealing can control the orientations of crystals that are produced, which may be crucial for certain purposes, such as nonlinear optics. Thermal annealing and rapid photothermal annealing are the two most common annealing techniques utilized for semiconductor optical fibers [103,104]. To effectively grow large crystal grains inside fibers, the technique involves two steps. First, fibers are heated to a low temperature of approximately 530 °C for 3 days to control nucleation site numbers, and second, the fibers are heated to approximately 1300 °C for 10 min. Compared with the pre-annealed fiber, the resulting fibers exhibited a much lower dislocation density and optical loss [105]. In this case, crystal sizes were restricted to 100 µm. Typically, thermal annealing is performed to transform amorphous semiconductor core material generated by the HPCVD technique into crystalline material. Moreover, both the absorption spectra and temperature performance can be changed by thermal annealing, as the technique can shift the bandgap of semiconductors and enhance the PL intensity [106]. It has been observed that SiGe alloy fibers produced by thermal drawing were subsequently annealed in a box furnace with a controlled heating rate [58]. Due to grain-to-grain diffusion, the homogeneity of the fiber core and optical performance of the fiber were enhanced.

#### 2.3.2. Rapid Photothermal Processing

The rapid thermal processing (RTP) technique that is dominated by quantum effects is referred to as RPP. RPP allows semiconductor devices to be treated at temperatures that are lower than those required by RTP. As a result, RPP can produce semiconductor optical fibers with improved performance. The high throughput of RPP, in conjunction with its low processing temperature, offers new possibilities for devices such as solar cells, silicon integrated circuits, and flat panel displays [107]. Photons with wavelengths of less than approximately 400 nm are essential to witness quantum phenomena. Quantum effects have several positive consequences for RPP. First, the bulk and surface diffusion coefficients are increased. Second, less time is spent in each cycle of processing. Third, there is a significant decrease in the number of microscopic flaws, which results in improved performance [107,108]. In the case of RPP, the bulk and surface diffusion coefficients are greater than those of RTP and standard furnace processing. Additionally, quantum photo effects have a crucial role in ion implantation [109]. In regard to performance, reliability, and yields, RPP will deliver a significant improvement over other production processes. RPP makes available materials that have consistent microstructures and built-in dependability [108].

#### 2.3.3. Laser Treatment

Laser treatment of semiconductors inside optical fibers is a very adaptable technique that can be utilized to change the semiconductor material in a variety of ways. For instance, CO_2_ laser processing has been utilized to heat fibers and recrystallize fiber cores, thereby resolving the uneven segregation issue of semiconductor alloys during nonequilibrium solidification and improving optical transmission behavior [59] by accurately regulating the heating temperature and cooling speed to produce single-crystal semiconductor materials [77]. This technique allows the fabrication of semiconductor core fibers of any length with low loss. Similar techniques have been employed to reduce the optical loss of germanium core fibers [110]. In contrast to conventional annealing techniques, the semiconductors of fiber are regionally heated to a molten state and then recrystallized under precise control as the fiber is scanned past the laser’s focal point [93]. Unlike tapering methods, which must equally heat the core and cladding materials, laser processing allows for selective heating of either the core or the cladding, depending on the selected wavelength.

Two primary heating methods have emerged concerning laser treatment. The direct optical absorption of the laser beam was the first method shown to successfully heat and melt the core. A laser source with photon energy larger than the semiconductor’s electronic bandgap is utilized since this kind of laser can pass through the cladding material but is significantly absorbed by the core through electronic absorption. By adjusting the scan rate, we may manipulate the strain and the electronic bandgap of the material. The second method involves the use of heat carried from the cladding to melt the semiconductor. For silica cladding fibers, CO_2_ lasers are commonly employed as the source since they can be absorbed by the cladding material. This technique allows for the development of single crystals with cm-scale lengths and high aspect ratios with minimal strain [77,111]. Both methods may be used in combination, with energy being absorbed by the core and cladding in a single manufacturing step.

#### 2.3.4. Interfacial Modifier

The utilization of an intermediate layer between the semiconductor core and the silica cladding can reduce tension, expel pollutants, and establish a gradient index of refraction [112]. A suitable interfacial material should meet the following guidelines. First, the material should be an oxide to reduce chemical sophistication; second, the material should form a eutectic with a semiconductor inside the fiber and possess a softening point that is higher than the semiconductor material to alleviate tension; last, the metal ion of the oxide should be less electronegative than the semiconductor to aid oxygen scavenging from the core [60]. Calcium oxide meets the aforementioned characteristics and is often employed in the purification of silicon [113], similar to other alkaline earth oxides. CaO reacts with water to create hydroxides; hence, a preform with a CaO layer on the interior of the silica may be prepared using an aqueous process. Further studies of NaO, BaO, SrO, and MgO were conducted with varying degrees of success: SrO and NaO, as well as NaO-BaO mixes, were the least successful, leading to deformation of the glass–silicon interface or immense voids in the core, while CaO and CaO-MgO blends behaved the best. Prior research mostly focused on the mechanical and microstructural qualities of the material. The optical characteristics of the fibers may be enhanced by using more pure raw materials [60].

## 3. Main Structures for Semiconductor Optical Fibers

**Figure 5:**
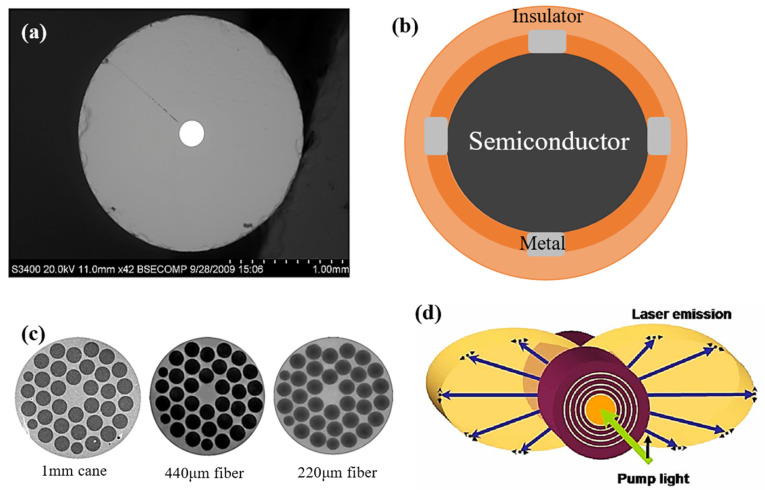
Semiconductor optical fibers of different geometries: (**a**) semiconductor core fiber (Reprinted with permission from ref. [119]. Copyright 2010 Optical Society of America), (**b**) metal-semiconductor-insulator fiber, (**c**) MOFs (Reprinted with permission from ref. [120]. Copyright 2003 Optical Society of America), and (**d**) PBG fiber (Reprinted with permission from ref. [121]. Copyright 2006 Optical Society of America).

We have thus far described several fabrication techniques and postprocessing methods for semiconductor multimaterial optical fibers. In this section, we will discuss the most common structures for semiconductor optical fibers, including semiconductor core fibers, metal-semiconductor-insulator fibers, MOFs, and PBGs. The geometries of the semiconductor optical fibers are shown in Figure 5.

### 3.1. Semiconductor Core Fiber

Semiconductor core fibers may combine nonlinear, Raman, and IR transparency of semiconductor planar waveguides with the advantages of long, elastic, and resilient fiber-based waveguides [114]. The most frequently employed technique for fabricating optical fibers that contain semiconductor cores is the MCM, as mentioned in the former section. For the MCM to work, the cladding glass has to soften and draw at temperatures higher than the core’s melting point. Initial measures employed silica glass as a cladding material for silicon core fiber, whereas DURAN, an alkaline borosilicate glass, was utilized for germanium core fiber. Silicon melts at approximately 1414 °C, while silica crystallizes at approximately 1950 °C. Although it would be preferable if the draw temperature more closely matched the melting point of the core, silica is commercially accessible and has high strength. For germanium and DURAN, a superior thermal match is achieved, as germanium melts at approximately 938 °C, whereas DURAN melts at approximately 1000 °C. After fiber fabrication, oxygen from cladding glass is detected in the fiber core, and its concentration increases with decreasing core size [114]. The oxygen concentration of the germanium core is much lower than that of the silicon core. The main reasons for the concentration difference can be described as follows. First, the higher temperature of silicon core (1950 °C) fiber thermal drawing activates more oxygen dissolution than germanium (1000 °C) [115]. Second, at high temperatures, germanium oxide is volatile [116,117]. In this instance, germanium oxidation melt produces a volatile product that, given the low viscosity of the germanium melt [118], should diffuse from the molten core.

### 3.2. Metal-Semiconductor-Insulator Fiber

Most practical electrical and OE devices require a combination of conductors, semiconductors, and insulators with well-defined geometries and at stipulated length scales while generating intimate surfaces [24]. Typically, the fibers are manufactured utilizing a variety of complicated wafer-based methods, which permit microscopic features but are constrained to planar geometries and restricted covering areas [122]. In comparison, the procedure of pulling fibers from a prefabricated reel or tube is easier and produces longer, highly uniform fibers with controlled geometries and excellent optical transport characteristics. This method has thus far been limited to certain materials [123] and larger features [124]. Thermal drawing can be utilized to fabricate a fiber photodetector with a core of amorphous semiconductors surrounded by metallic microwires. Such a fiber is sensitive to light over its whole length (tens of meters), constituting a one-dimensional photodetection element. The fiber can be woven to design a grid of fibers that are capable of identifying the position of an illumination point.

The variety of metal-semiconductor-insulator fiber structures enables fibers to possess various functions. A fiber that integrates optical, electrical, and thermal components can act as a self-monitoring optical transport device [125]. To direct high-power radiation along the fiber axis [126], hollow-core multilayer cylindrical PBGs [127,128] are utilized in the fiber transmission element. To keep track of the temperature throughout the length of the fiber, long metal wires connected at their ends by a semiconductor layer respond to changes in the fiber’s temperature by sending a current to the fiber’s terminals. Self-monitoring, high-power optical transmission lines can predict and prevent failures because the electrical conductivity of the semiconducting material exponentially depends on temperature, allowing for instantaneous discrimination between normal transmission conditions and those indicative of localized defect formation [125].

### 3.3. Microstructured Optical Fiber

MOF is an innovation that is characterized by a series of nanoscale capillaries to microscale capillaries. These capillaries are designed in geometries to guide light via photonic bandgap effects [129,130]. Physical methods such as drawing and fluid infiltration are being used to include gases [131], liquid crystals [132], polymers [24], and low-melting-point solids, including metals [133,134], compound glasses [25], and semiconductors in MOFs, hence increasing the versatility and utility of fiber devices [123,135]. High-performance semiconductors and junctions are indispensable for use in high-performance OE and photonics [136]. Transistors, light-emitting diodes/lasers, and high-speed photodetectors rely on p-n junction creation, which is enabled by carefully managed impurity doping.

MOFs can be deposited by the HPCVD technique, which can integrate conformal semiconductor layers and void-free wires inside micro-to-nanoscale-diameter pores [95]. The use of fibers as templates for the manufacturing of these semiconductor devices enables more geometric design freedom than is possible with planar fabrication. Thus, all-fiber technical approaches are realized by enabling the seamless integration of OE and photonic devices with fiber infrastructure. Junction-based fiber devices with gigahertz bandwidth, for instance, can be manufactured from doped crystalline semiconductors [95]. As it is impossible to draw on amorphous hydrogenated silicon, its nonlinear optical properties may be exploited by fiber deposition. Ultimately, the cores of fibers fabricated from crystalline compound semiconductors show promise for high-power infrared, light-guiding fiber devices and subwavelength-resolution, wide-area infrared imaging.

In the procedure, pressures ranging from 8 to 35 megapascals are applied to MOF pores to force chemical precursor mixtures, such as silane and dopants in a gas mixture of helium or carbon dioxide, to flow through the MOF holes. At temperatures that have been properly selected, the precursor breakdown, layer deposition, and dopant integration processes take place within the pores. Amorphous semiconductors, such as Ge, may be deposited at lower temperatures using group IV hydride precursors. Polycrystalline semiconductors, such as crystalline Si tubes, can be formed by annealing amorphous materials at elevated temperatures [123].

### 3.4. Photonic Bandgap Structured Fiber

PBGs, commonly referred to as photonic crystals (PCs), emerged from an original concept by Eli Yablonovitch [137]. For PBG materials, the propagation of photons in a certain energy range is likewise prohibited. On a length scale comparable to optical wavelengths, PBGs exhibit an alternative refractive index [137]. PBGs may be classed as 1D, 2D, or 3D. 1D PBGs are generally composed of alternating dielectric layers with alternative refractive indices. 2D PBGs may be planar structures having a periodic pattern in two dimensions or PBG fibers [138], for example, the hollow core, or depressed index solid core PBG fibers, where a PBG is in the cladding. 3D PBGs have a periodic refractive index in three dimensions and often include the opal or inverse opal type.

An omnidirectional dielectric mirror fiber was fabricated by thermal drawing from a multilayered fiber preform [20]. Glassy materials with very different refractive indices but equivalent thermomechanical capabilities were employed to create 21 layers of alternating refractive indices that surrounded a durable polymer core. These fibers might be used in a variety of contexts, such as woven radiation barriers, spectral identification of cloth, and telecommunications filters. Dielectric mirror fibers that act as omnidirectional mirrors may redirect light from any direction and of any polarization that strikes them. Altering the layer thickness by just a few atomic layers may produce transmission spectra that span the electromagnetic spectrum. The transmission peaks in the visible, ultraviolent (UV), near-infrared (NIR), and middle infrared (MIR) sections of the optical spectrum for fibers with various layer thicknesses. Due to the modest material absorption, multimaterial hollow-core, PBG optical fibers may provide lasers with low transmission loss at peak outputs of 11.4 MW at 1.55 μm, with 97% of the fiber output in the fundamental mode [139].

## 4. Potential Biomedical Application

### 4.1. Thermoelectric Fiber for Human Body Temperature Regulation

**Figure 6:**
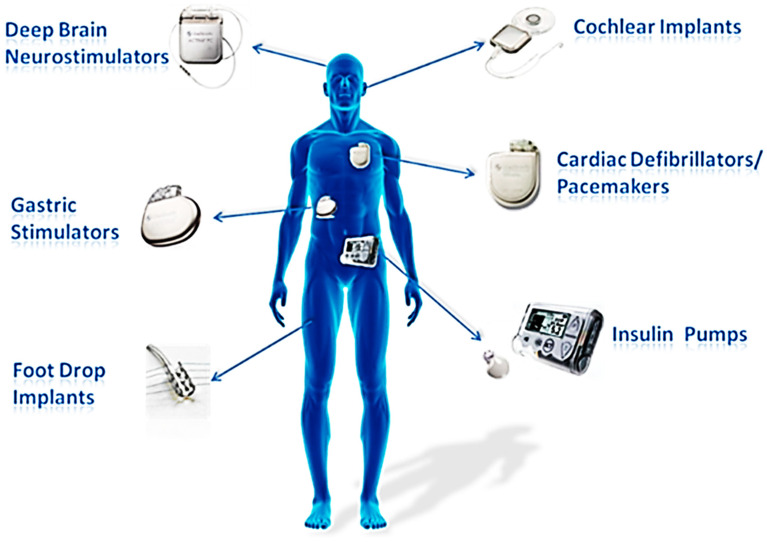
Common semiconductor implantable devices. (Reprinted with permission from ref. [168]. Copyright 2019 PM Kumar et al.).

Based on the Peltier effect and Seebeck effect, the applications of TE devices may be categorized into two groups: fiber-based thermoelectric coolers (FTECs), which transfer electrical energy into heat, and fiber-based thermoelectric generators (FTEGs), which transfer heat into electrical energy [140]. Popular inorganic TE materials are shown in Table 1. FTECs have features such as small volume and precise temperature control without vibration and noise. Despite their hard construction, these devices have been employed to replace more conventional methods of cooling, such as those used in refrigerators and electronic gadgets’ central processing units [141,142,143]. As a bonus, solar energy may be used by coolers in tandem with a heating system to both chill and warm an area throughout the summer and winter months [144]. The application of FTECs may decrease environmental pollution and climate change [145]. FTECs can accomplish this reduction because they are more adaptable and lightweight than conventional TECs, hence mitigating their disadvantages [146]. When the ambient temperature is greater than the normal skin temperature, a fabric system containing FTECs may maintain a cool microclimate. Consequently, such clothing is particularly effective for preventing heat stroke for persons who operate in high-temperature environments, such as construction workers in subtropical and tropical countries and steelworkers [140]. In addition, FTEGs have a vast promising application scope, ranging from space and military to autos, aircraft, biomedicine, and smart textiles, and can employ a variety of heat sources, such as biothermal and waste heat from transportation equipment. When astronauts conduct extravehicular activities in outer space, where the temperature ranges from −233~121 °C, they wear hermetic space suits [147]. Liquid cooling and ventilation are conventional space-suit thermal management methods. The ventilation unit or airflow duct on this clothing reduces its air permeability [148]. FTECs and FTEGs combine their benefits to solve the temperature and moisture comfort challenges of current space suits. When outer space is hotter than the body temperature, FTEGs will power FTECs [149]. This application can be further introduced to patient body temperature; common medical applications for semiconductor FTEGs are depicted in Figure 6.

In addition, the transformation of human body heat into electric power is one of the most significant uses of textile-based FTEGs. Human body heat is generally maintained at 37 °C and created via metabolic processes [150]. The most apparent benefit of the transformation is that energy can be collected regardless of activity and may be gathered from the whole body’s surface. Textile-based FTEGs may thus be utilized to support the functioning of small electronic devices, such as sensors and implantable medical devices (IMDs), such as cochlear implants, drug pumps, neurostimulators, muscle stimulators, and pacemakers. These gadgets only need a few microwatts to milliwatts of power to operate [151,152,153]. Since the introduction of the first implantable medical device in 1972, IMDs have been extensively utilized for diagnosis, prognosis, and therapy [154]. One difficulty in the development of IMDs is that their batteries, the device’s energy source, may reach the end of their useful life after prolonged use [155]. A reliable and continuous power source is essential to sustain the functionality of IMDs to prevent needless surgery and patient costs. Consequently, the use of FTEGs to power IMDs is a viable solution to this issue.

**Table 1:** Popular inorganic TE materials.

Inorganic TE Materials		Type	ZT	Temperature(K)	Reference
Half-Heusler compound	Hf_0.6_Zr_0.4_NiSn_0.98_Sb_0.02_	n-type	≈1.0	1000	[156]
	Zr_0.5_Hf_0.5_CoSb_0.8_Sn_0.2_	p-type	0.8	973	[157]
	Hf_0.8_Ti_0.2_CoSn_0.8_Sn_0.2_	p-type	1.0	1073	[130,158]
Bi-Te alloy	FeNb_0.86_Hf_0.14_Sb	p-type	≈1.5	1200	[159]
	Bi_x_Sb_2-x_Te_3_	p-type	1.4	373	[160]
	Bi_0.3_Sb_1.7_Te_3_	p-type	1.33	373	[161]
	Bi_0.5_Sb_1.5_Te_3_	p-type	1.25	320	[162]
	Bi_2_Te_2.79_Se_0.21_	n-type	1.2	357	[163]
Skutterudite compounds	Co_3.2_Fe_0.8_Sb_12_	p-type	0.53	823	[164]
	Ca_0.31_Co_4_Sb_12_	p-type	1.15	840	[165]
	CeFe_4_Sb_11.9_Te_0.1_	p-type	0.76	773	[166]
	La_0.75_Pr_0.25_Fe_4_Sb_12_	p-type	0.83	823	[167]

### 4.2. Optoelectronic Fibers for Sensing the Human Body’s Interior

**Figure 7:**
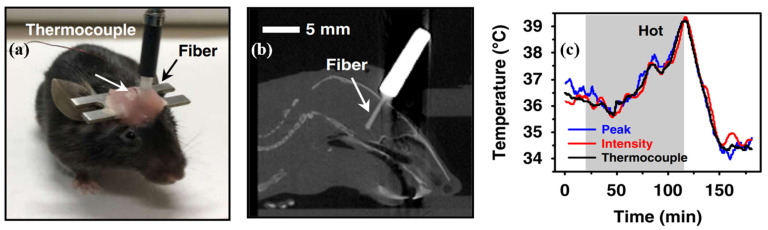
OE fiber-integrated devices for in vivo temperature sensing (Reprinted with permission from ref. [180]. Copyright 2022 He Ding et al.): (**a**) a mouse implanted with a fiber sensor for temperature detection; (**b**) sagittal CT slice reconstruction; and (**c**) dynamic temperature data acquired by the fiber sensor in the mouse brain.

One typical use for optical fibers is in the field of optical detection. OE qualities are necessary for multimaterial fiber photodetectors that are used in medical applications, such as internal observation and imaging. Various semiconductors possess a wide range of characteristics, making semiconductor OE fibers appealing [169]. These characteristics include a broad range of infrared transparency, significant optical nonlinearity, and a high refractive index [66]. All examples of nonlinear behavior in semiconductor optical fibers have been linked with centrosymmetric crystal systems, with the major portion associated with the silicon fiber framework [93]. The transformation of optical power into electrical power is the fundamental process that underpins the majority of semiconductor-detecting or energy-collecting OE devices [170]. This transformation is a significant advantage of the semiconductor OE fiber platform over other similar technologies. Both in-fiber detectors and “solar threads” have been manufactured using this technology to achieve this goal [102,123]. This platform is distinguished from previous fiber technologies by the possibility of incorporating OE functionality into the constituent material of the waveguiding fiber. Moreover, the OE property can be further utilized to detect in vivo temperature, as demonstrated in Figure 7. The fiber-integrated device can measure deep brain temperature variations. Complementary to tethered electrical sensors, this optical-based approach is more appropriate for use in areas with significant electromagnetic interference and is especially capable of acquiring data during magnetic resonance imaging (MRI). As a result, various fiber optic sensors based on OE characteristics, such as the bandgap of a GaAs crystal material [171], have been implemented [172,173].

The most common geometry for multimaterial OE fibers is a metal-semiconductor-insulator fiber, as mentioned in the former section. Within a cladding constructed of transparent thermoplastic, the earliest kinds of OE fibers combined metals, polymer composites, and chalcogenide semiconductors [66]. Chalcogenide glasses may be produced either by starting with the chalcogen elements S, Se, and Te or by adding additional elements such as As, Ge, Sn, or Ga [174]. The chalcogenide semiconductors integrated in OE fiber structures include As_40_Se_50_Te_10_Sn_5_ [24], Se_97_S_3_ [175], SnZn [131], and ZnSe [176]. The OE fibers are fabricated mainly through thermal drawing and HPCVD.

The first metal-semiconductor-insulator OE fiber was produced by thermal drawing [24]. The photoconducting fiber could be employed to detect incoming light on the fiber’s surface along its length. After weaving these fibers into grids, it is possible to determine the phase and amplitude of an electromagnetic wave across wide regions for lensless-free imaging systems [177]. Within the confines of a single fiber, it was possible to create cascading thin films that combined several photodetection devices with feature sizes as small as 100 nanometers. The fiber was able to distinguish between two wavelengths in the visual range with a precision of less than 5 nm and an angular resolution that was accurate to within 4 degrees [178]. In addition, the assembly of these fibers into two-dimensional grids produced textiles that were able to both localize a point of light and carry out sophisticated optical tasks, such as the imaging of an object without the need for lenses [177,178]. The fiber’s infrared OE capability and adaptability enable it to function as an in vivo sensor to identify anomalies in human tissues and organs.

Utilizing HPCVD is another option for the monolithic incorporation of metals and semiconductors in fibers. Multiple devices, including ohmic contacts [66], p–n junctions [179], Schottky junctions [123], and p–i–n junctions [102], may be manufactured due to the sequential deposition of various materials. The Pt/n-Si Schottky junction that was manufactured using the HPCVD junction had a barrier height of 0.8 eV and displayed a 3 dB bandwidth with a maximum of 3 GHz at a wavelength of 1550 nm. These dimensions allowed photodetection to be performed at telecommunication wavelengths within a limited amount of time [123]. A polycrystalline silicon p–i–n junction is successfully achieved across a length of one meter of silica MOF. When lit across a length of 1 mm and subjected to 100 MHz waveguide photodetection at 1064 nm, the fiber had a bandwidth that reached 1.8 GHz, which was 3 dB wide [102]. Due to its increased quantum efficiency, this fiber is advantageous for its use in applications relating to photodetection and photovoltaics. Under the conditions of air mass 1.5 solar illumination, the total conversion efficiency of a 1 cm p–i–n fiber that was electrically connected on both ends showed a value of 0.5%. This fiber is effective for photodetection due to its high quantum efficiency and may be incorporated into medical equipment for lesion exploration during gastrointestinal and colonoscopy surveillance.

### 4.3. Gas Sensor for VOC Detection

Disease-related gas detection is receiving growing medical interest. In healthy individuals, the amounts of VOCs in exhaled breath are often detected at subppm values or even lower values. Even if the association between a particular illness and certain VOCs has been recognized for more than a century, only current technologies based on semiconductor gas sensors can provide the quantitative measurements required for stringent clinical practice. Clinical experiments have shown the potential for utilizing breath to diagnose major diseases, including many forms of cancer, diabetes, multiple sclerosis, and kidney disease [181]. The invention and widespread use of solid-state gas sensors have offered an additional boost to the interest in VOC inspection and analysis for medical applications [182]. Semiconducting gas sensors are employed to test certain gases, such as hydrogen peroxide [183], acetone [184], ammonia [185], nitric oxide [186], and volatile sulfur [187], from human bodies. However, the study is being undertaken to create detection systems based on commercial MOX detectors to monitor low quantities of VOCs in breath [188], which is further enhanced by temperature modulation [189]. Particular breath biomarkers not only identify the existence of particular illnesses but also represent the overall physical state. An example of such gadgets is mobile acetone analyzers that monitor the ketone level and determine body fat burning rate only from exhaled breath. The devices that track fat metabolism and ketosis levels are widely utilized in a variety of diet and exercise regimens. SnO_2_ nanofibers functionalized with reduced graphene (rGO) that were assessed for the identification of acetone and hydrogen sulfide in human breath are an example of the utilization of semiconductors in breath analysis [190].

**Figure 8:**
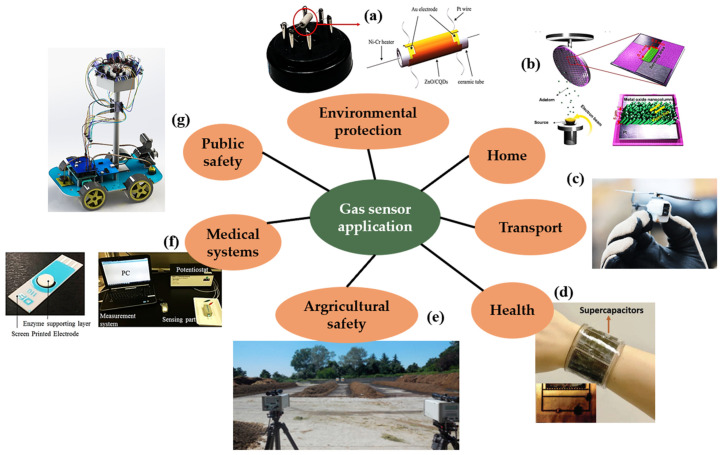
Semiconductor gas sensor applications. (Reprinted with permission from ref. [194]. Copyright 2020 MV Nikolic et al.): (**a**) NO gas sensor for environmental protection; (**b**) metal-oxide nanocolumns sensor for fire detection; (**c**) smelling nano aerial sensor for gas source localization and mapping; (**d**) self-powered sensor wristband for health and fitness applications; (**e**) ammonia emissions measured using GasFinder open-path lasers; (**f**) ethanol vapor detection gas sensor for medical systems; and (**g**) multiple hazard gas detector for air quality monitoring.

With health care applications, gas sensors are utilized for wearable biosensors. Wearable biosensors are small, complicated devices that have wireless connection modules for sending sensor data to computational infrastructure [191]. In such sensing devices, many chemicals are utilized. In the case of semiconducting oxide materials, this use presents a problem since the working conditions of the sensor must be lowered to room temperature. Nanostructures, such as graphene or nanowires, are one technique to attain this goal [192]. Recently, a stretchy and flexible, self-heating metal oxide (MOx) gas-sensing platform was developed [193]. Compared with their industrial equivalents, the development of wearable gas sensors must meet stringent criteria, such as a compact and lightweight form factor, low working temperature, low energy consumption, and mechanical durability upon diverse skin deformations. Latest applications of gas sensors are depicted in Figure 8.

### 4.4. Fiber Lasers for Medical Surgery

**Figure 9:**
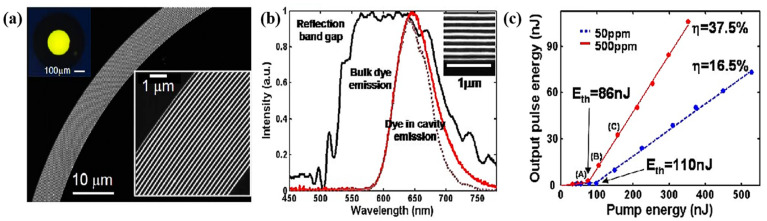
PBG fiber laser (Reprinted with permission from ref. [121]. Copyright 2006 Optical Society of America): (**a**) Fiber structure; (**b**) Fluorescence spectrum shown in red, and measured reflection band gap shown in black; (**c**) Dependence of the laser energy on the pump energy.

All fiber lasers that have been manufactured have been designed to emit light down the axis of the fiber, with the spot size determined by the core radius and the wavefront being virtually planar [195]. The cavity that is necessary for lasing is created by reflection off of the end facets of the fiber. There are numerous applications in which it is advantageous to deliver laser light in the radial direction over an extensive area [196]. Some examples of these applications include textile fabric displays [197] and biomedical applications ranging from photodynamic therapy [198] to in vivo molecular imaging [199]. A radial lasing structure [121] is produced as a consequence of omnidirectional reflection originating from an annular multilayer mirror that is lined inside of a hollow-core fiber. As shown in Figure 9, the fiber allows for the transmission of a pulse optical pump at 532 nm and covers the complete fluorescence spectrum (Figure 9b). Moreover, the dependency of energy on pump energy is shown for both 500 ppm and 50 ppm dye concentrations (Figure 9c). The slope efficiencies for the 500 ppm and 50 ppm concentrations are 37.5% and 16.5%, respectively.

Omnidirectional dielectric mirror fibers can reflect light incidence in any direction, independent of the angle or polarization. A minute modification to the layer thickness on the nanoscale scale may produce the spanning transmission spectra that are desired. The transmission of light through fibers that have varied layer thicknesses peaks in the visible, ultraviolet, near-infrared, and mid-infrared parts of the optical spectrum. Due to the low material absorption, multimaterial, hollow-core, PBG optical fibers are capable of delivering lasers with minimal transmission loss, with 97% of the fiber output occurring in the fundamental mode [139]. The transmission of high-peak-power laser pulses in highly controlled spatial patterns through fiber optics provides the foundation for a wide variety of medicinal and industrial applications [139]. Fibers with fundamental PBGs at 350 nm (UV) and 750 nm (NIR) have been utilized for biomedical purposes, whereas fibers with fundamental PBGs at 10.6 μm (MIR) have been used for laser surgery [195]. Because of the MIR wavelength laser’s strong water absorption at 2.9 μm in human tissue and small surgically damaged area [200], it is suitable for use in a wide variety of medical and surgical applications, including otorhinolaryngology, gynecology, neurosurgery, urology, dermatology, ophthalmology, dental surgery, and cardiovascular surgery [201,202]. The use of the CO_2_ laser in neurosurgery is superior. Because of its high water absorption rate, the CO_2_ laser generates minimal heat and protects the surrounding tissue [203,204]. There have been commercial fiber lasers for medical surgery. Semiconductor saturable absorber mirrors (SESAMs) with a quantum well structure placed on Bragg mirrors are the most developed and commonly used SAs in available commercial ultrafast lasers [205]. However, the intricate fabrication procedure of SESAMs utilizing molecular beam epitaxy renders them rather costly and rigid.

## 5. Conclusions and Outlook

In this paper, a review of semiconductor multimaterial fibers was presented with respect to materials, fabrication methods, device structures, and applications. First, we take into account the manufacturing processes that are most commonly utilized to produce semiconductors and various methods that may be used to fabricate semiconductor multimaterial fibers, followed by post-fabrication procedures that improve the functionality of semiconductor optical fibers. Second, we discuss semiconductor optical fibers with different structures. Last, we describe the innovative functions that can be achieved by integrating semiconductors into fibers and their potential applications in medical and surgical procedures.

The developments of novel fiber materials and compositional structuring are key themes that arise from current works in studies on semiconductor optical fibers. The examination of the kinetics during fiber manufacture and postprocessing techniques generates positive effects on significant improvements in performance. Many different topics warrant additional research, the most important of which is the role of cladding-induced stress in determining whether clean grain boundaries are detrimental to optical applications. In addition, an exploration to limit the integration of impurities in fabricated fiber or remove impurities from fabricated fiber is important [51,119,206,207].

Although multifunctional fibers have had to rely on inorganic chalcogenide glasses for their OE functionality and simplicity of fabrication, it is anticipated that by provoking phase transitions [208,209,210], semiconductor crystalline domains with outstanding electronic properties can be incorporated within fiber devices. The use of OE and logic processes may be facilitated by the use of electronic junction structures, such as p-n junctions and Schottky junctions. Numerous additional qualities contribute to the usefulness of semiconductors. For example, the Kerr nonlinearity of As2Se3 is approximately three orders of magnitude larger than that of silica glass [211,212,213,214,215]. Therefore, the appearance of nonlinear optical phenomena predicated on these multimaterial glass fibers should be anticipated. Some examples of these effects include supercontinuum generation [216,217] and Raman amplification [218]. Other qualities include high magneto-optic coefficients [219]; these properties easily propose a vast variety of small fiber-based devices. Exciting new gadgets are on the horizon as more progress in the incorporation of electrical functionality into these fibers is achieved.

Fabrics woven entirely or in part from integrated fibers have the potential to deliver a wide variety of real-time, novel functionalities across the entire surface area of clothing. These functionalities are energized by electrical energy collected from the surrounding environment. The interaction between the characteristics of the materials and the integration of the structure in these fibers, coupled with the production of the fabric array, is emergent and has the potential to be an attractive subject for biomedical applications. Further advancement of transistors and fiber-integrated devices has the potential to produce increasingly more complex features and genuinely multifunctional textiles [195].
